# Effects of the Angiotensin Receptor Blocker Olmesartan on Adipocyte Hypertrophy and Function in Mice with Metabolic Disorders

**DOI:** 10.1155/2014/946492

**Published:** 2014-06-02

**Authors:** Akinobu Maeda, Kouichi Tamura, Hiromichi Wakui, Masato Ohsawa, Kengo Azushima, Kazushi Uneda, Tomohiko Kanaoka, Ryu Kobayashi, Kohji Ohki, Miyuki Matsuda, Yuko Tsurumi-Ikeya, Akio Yamashita, Yasuo Tokita, Satoshi Umemura

**Affiliations:** ^1^Department of Medical Science and Cardiorenal Medicine, Yokohama City University Graduate School of Medicine, 3-9 Fukuura, Kanazawa-ku, Yokohama 236-0004, Japan; ^2^Department of Molecular Biology, Yokohama City University Graduate School of Medicine, 3-9 Fukuura, Kanazawa-ku, Yokohama 236-0004, Japan; ^3^Renal Division, Department of Medicine, Fujisawa Municipal Hospital, 2-6-1 Fujisawa, Fujisawa 251-8550, Japan

## Abstract

In the present study, we examined the therapeutic effects of olmesartan, an angiotensin II (Ang II) type 1 receptor (AT1R)-specific blocker, in genetically obese diabetic KKAy mice, a model of human metabolic disorders with visceral obesity, with a focus on an olmesartan effect on the adipose tissue. Olmesartan treatment (3 mg/kg per day) for 4 weeks significantly lowered systolic blood pressure but did not affect body weight during the study period in KKAy mice. However, there were three interesting findings possibly related to the pleiotropic effects of olmesartan on adipose tissue in KKAy mice: (1) an inhibitory effect on adipocyte hypertrophy, (2) a suppressive effect on IL-6 gene expression, and (3) an ameliorating effect on oxidative stress. On the other hand, olmesartan exerted no evident influence on the adipose tissue expression of AT1R-associated protein (ATRAP), which is a molecule interacting with AT1R so as to inhibit pathological AT1R activation and is suggested to be an emerging molecular target in metabolic disorders with visceral obesity. Collectively, these results suggest that the blood pressure lowering effect of olmesartan in KKAy mice is associated with an improvement in adipocyte, including suppression of adipocyte hypertrophy and inhibition of the adipose IL-6-oxidative stress axis. Further study is needed to clarify the functional role of adipose ATRAP in the pleiotropic effects of olmesartan.

## 1. Introduction

Recently, metabolic disorder with visceral obesity has come to be recognized as a major medical condition related to significantly increased risks of hypertension, type 2 diabetes, dyslipidemia, and ultimately life-threatening cardiovascular disease [1]. Accumulating evidence also indicates that adipose tissue functions as a distinct endocrine organ capable of producing adipokines, such as adiponectin and leptin [2]. Furthermore, dysregulation of adipose tissue function is suggested to be closely involved in the pathophysiology of metabolic disorders via the stimulated production of inflammatory cytokines and upregulation of oxidative stress [3–5].

The renin-angiotensin system (RAS) plays an important role in the maintenance of circulatory and water-electrolyte homeostasis based on the generation of angiotensin II (Ang II), a potent vasoactive peptide, and the pathological activation of RAS has been implicated as one of the major contributors to hypertension and cardiovascular disease. Recent evidence has also indicated an important role of adipose tissue RAS in the physiological regulation of adipose tissue function, further suggesting a specific pathophysiological link between the dysregulated activation of adipose tissue RAS and the development of metabolic disorders and their complications [6, 7]. The physiological and pathophysiological actions of Ang II are principally mediated by the Ang II type 1 receptor (AT1R). In the present study, we examined the therapeutic effects of olmesartan, an AT1R-specific blocker, in genetically obese and diabetic KKAy mice, a model of human metabolic disorders with diabetes without any dietary loading [8], and focused our analysis on adipose tissue.

## 2. Materials and Methods

### 2.1. Animals and Treatment

C57BL/6 mice (male) and KKAy mice (male) were purchased from CLEA Japan, Inc. (Tokyo, Japan) for use as a nondiabetic normal control and a model of metabolic disorders with type 2 diabetes, respectively [9–11]. These mice were housed in a controlled environment with a 12 h light-dark cycle and were allowed free access to food and water. They were fed a standard diet (3.6 kcal/g; 13.3% energy as fat; Oriental MF, Oriental Yeast, Co., Ltd.). Male KKAy mice at 9 weeks of age were treated with the oral administration of olmesartan (3 mg/kg per day) in drinking water for 4 weeks, and body weight and food intake were measured. The KKAy mice treated with vehicle were previously described [12]. On the other hand, C57BL/6 control mice were treated with vehicle during the study period. Mice were sacrificed under anesthesia and the tissues were collected at the end of the experimental period. The protocol was reviewed and approved by the Animal Studies Committee of Yokohama City University and all experiments were performed in accordance with the National Institutes of Health guidelines for the use of experimental animals.

### 2.2. Blood Pressure Measurement by Tail-Cuff Method

Systolic blood pressure was measured noninvasively by the tail-cuff method (BP-monitor MK-2000; Muromachi Kikai Co.). The MK-2000 BP-monitor allowed determination of the blood pressure without any preheating of the animals, thus avoiding this very stressful condition [13–15]. At least eight readings were taken for each measurement.

### 2.3. Preparation of Tissue Sections and Histological Analysis

The epididymal white adipose tissue was isolated and fixed with 10% paraformaldehyde overnight and embedded in paraffin. Tissue sections were stained with hematoxylin and eosin for cell size determination. The adipocyte diameter and area were quantified using Image-Pro Plus software.

### 2.4. Tissue RNA Isolation and Real-Time Quantitative Reverse Transcript-PCR (qRT-PCR) Analysis

Total RNA was extracted from epididymal adipose tissue with ISOGEN (Nippon Gene), and cDNA was synthesized using the SuperScript III First-Strand System (Invitrogen). Real-time qRT-PCR was performed with an ABI PRISM 7000 Sequence Detection System by incubating the reverse transcription product with TaqMan PCR Master Mix and a designed TaqMan probe (Applied Biosystems), essentially as described previously [15–18]. The mRNA levels were normalized to those of the 18S rRNA control.

### 2.5. Statistical Analysis

All data are shown as mean ± SEM. Differences were analyzed by ANOVA followed by the Newman-Keuls multiple-comparison test. A *P* value of <0.05 was considered statistically significant.

## 3. Results

### 3.1. Effects of Olmesartan on Blood Pressure, Body Weight, and Food Intake

**Figure 1:**
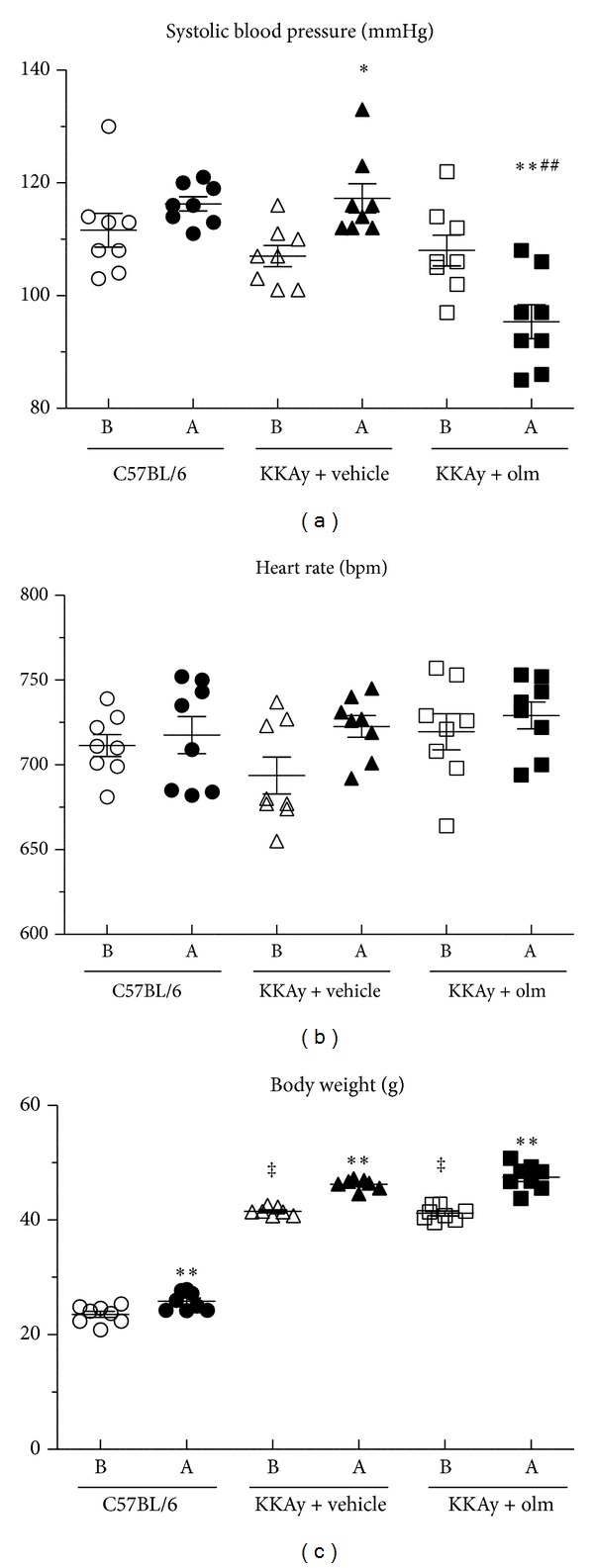
Effects of olmesartan (olm) on systolic blood pressure (a), heart rate (b), and body weight (c) in KKAy mice. Individual values are shown in the graphs and the values are also shown as the mean ± SEM (*n* = 8). B, before treatment; A, after treatment. *P < 0.05, **P < 0.01 versus before treatment; ^##^ *P* < 0.01 versus KKAy + vehicle; ^‡^ *P* < 0.01 versus C57BL/6 (ANOVA).

The systolic blood pressure, heart rate, and body weight at baseline and after the study period in the control C57BL/6, vehicle-treated KKAy, and olmesartan-treated KKAy mice are shown in Figure 1. At baseline there were no significant differences in systolic blood pressure or heart rate between the groups before the treatment. Body weight at baseline was significantly greater in the KKAy mice of either treatment group than in the control C57BL/6 mice (*P* < 0.01 versus C57BL/6). With respect to the effects of treatment with olmesartan at a dose of 3 mg/kg per day for 4 weeks on heart rate and body weight, heart rate was not affected in the KKAy mice by the treatment with olmesartan for 4 weeks compared with baseline (baseline versus 4 weeks, 720 ± 11 versus 729 ± 8 bpm, NS), and there was a significant increase in body weight after the olmesartan treatment (baseline versus 4 weeks, 41.2 ± 0.4 versus 47.5 ± 0.8 g, *P* < 0.01). After 4 weeks, heart rate and body weight did not differ significantly between the vehicle-treated and olmesartan-treated KKAy mice (Figure 1).

**Figure 2:**
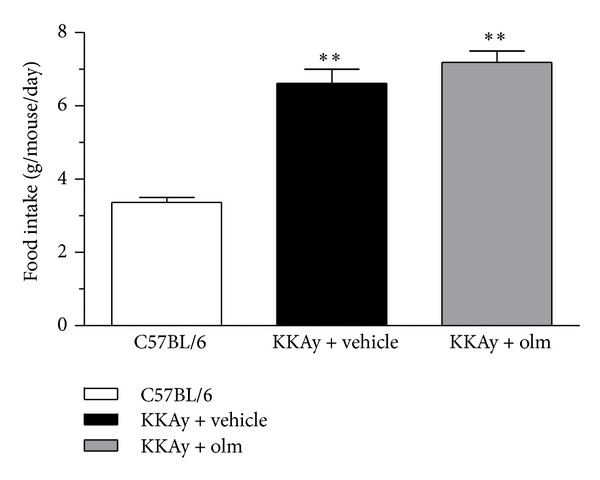
Effects of olmesartan (olm) on daily food intake in KKAy mice. The values are the mean ± SEM (*n* = 8). **P < 0.01 versus C57BL/6 (ANOVA).

On the other hand, systolic blood pressure in the KKAy mice was significantly decreased by the olmesartan treatment for 4 weeks compared with baseline (baseline versus 4 weeks; 108 ± 3 versus 95 ± 3 mmHg, *P* < 0.01), and systolic blood pressure was significantly lower in the KKAy mice treated with olmesartan than those treated with vehicle (*P* < 0.01 versus vehicle) (Figure 1). Furthermore, although the daily food intake after 4 weeks was significantly greater in the KKAy mice of either treatment group than the control C57BL/6 mice (*P* < 0.01 versus C57BL/6), the daily food intake was similar in the vehicle-treated and olmesartan-treated KKAy mice groups (Figure 2).

### 3.2. Effects of Olmesartan on Adipocyte Hypertrophy in KKAy Mice

**Figure 3:**
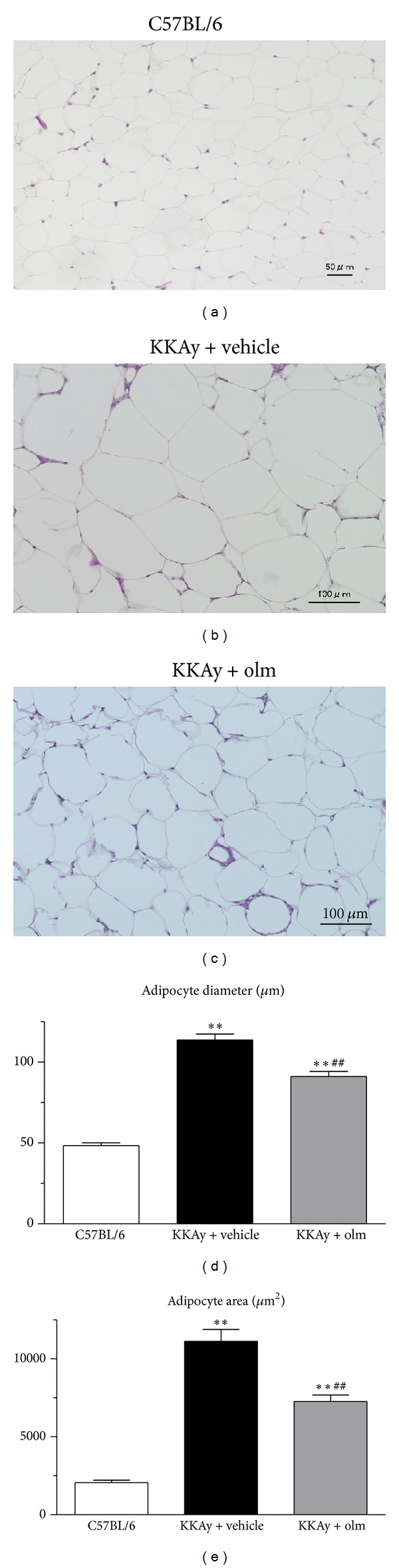
Effects of olmesartan (olm) on adipocyte hypertrophy in KKAy mice. Upper panel: histological analysis of epididymal adipose tissue sections ((a) C57BL/6; (b) KKAy + vehicle; (c) KKAy + olmesartan) stained with hematoxylin and eosin in each experimental group. Original magnification: ×200. Lower panel: adipocyte diameter (d) and area (e). The values are the mean ± SEM (*n* = 8). **P < 0.01 versus C57BL/6; ^##^ *P* < 0.01 versus KKAy + vehicle (ANOVA). Olm indicates olmesartan.

We examined whether there was any phenotypic alteration in the adipose tissue of the KKAy mice treated with olmesartan. Although the KKAy mice treated with vehicle had significantly larger adipocytes than the control C57BL/6 mice (*P* < 0.01 versus C57BL/6) (Figures 3(a), 3(b), and 3(c)), adipocyte hypertrophy was significantly inhibited in the KKAy mice treated with olmesartan for 4 weeks (Figure 3(d); vehicle-treated KKAy mice versus olmesartan-treated KKAy mice; diameter: 113.7 ± 3.7 versus 91.2 ± 3.1 *μ*m, *P* < 0.01; Figure 3(e), area: 11131 ± 765 versus 7264 ± 415 *μ*m^2^, *P* < 0.01).

### 3.3. Effects of Olmesartan on Adipokine and Adipose Tissue RAS Gene Expression

**Figure 4:**
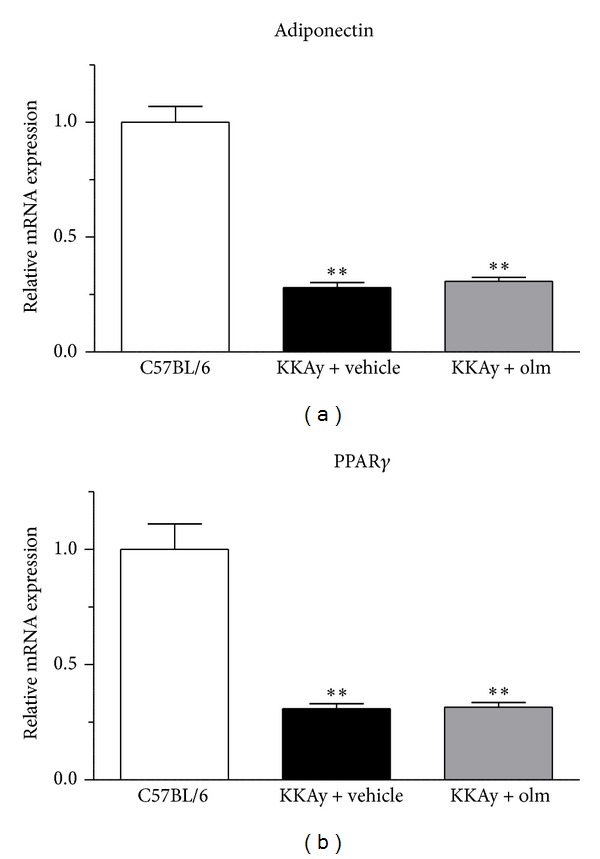
Effects of olmesartan (olm) on the adipose tissue mRNA expression of adiponectin (a) and PPAR*γ* (b) in KKAy mice. The values are the mean ± SEM (*n* = 8). **P < 0.01 versus C57BL/6 (ANOVA). Olm indicates olmesartan.

**Figure 5:**
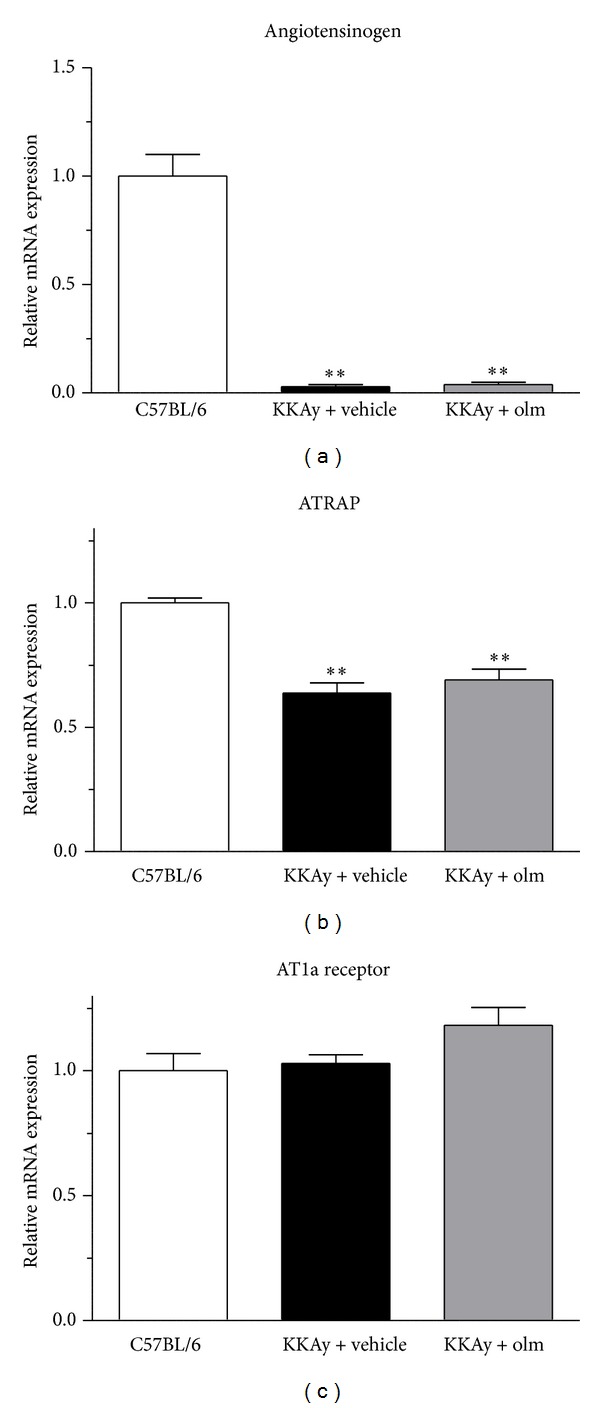
Effects of olmesartan (olm) on the adipose tissue mRNA expression of angiotensinogen (a), ATRAP (b), and AT1R (c) (AT1a receptor) in KKAy mice. The values are the mean ± SEM (*n* = 8). **P < 0.01 versus C57BL/6 (ANOVA). Olm indicates olmesartan.

As shown in Figure 4, the KKAy mice treated with vehicle exhibited significantly suppressed adipose tissue expression of adiponectin, an important adipokine, as well as peroxisome proliferator-activated receptor *γ* (PPAR*γ*), compared with the control C57BL/6 mice (Figures 4(a) and 4(b)). With respect to a possible effect of olmesartan on adiponectin and PPAR*γ*, the treatment with olmesartan did not affect adiponectin or PPAR*γ* mRNA expression in the adipose tissue of KKAy mice (Figures 4(a) and 4(b)). We also examined the possible influence of olmesartan on adipose tissue expression of the RAS component genes (angiotensinogen, ATRAP, and AT1R) in KKAy mice. While the KKAy mice treated with vehicle exhibited a significantly lower expression of adipose angiotensinogen and ATRAP mRNA than the control C57BL/6 mice (*P* < 0.01 versus C57BL/6), adipose AT1R mRNA expression was not altered in vehicle-treated KKAy mice (Figures 5(a), 5(b), and 5(c)). In addition, treatment with olmesartan did not affect the expression of angiotensinogen, AT1R, or ATRAP mRNA in the adipose tissue of KKAy mice (Figures 5(a), 5(b), and 5(c)).

### 3.4. Effects of Olmesartan on Adipose Tissue Inflammatory Cytokines

**Figure 6:**
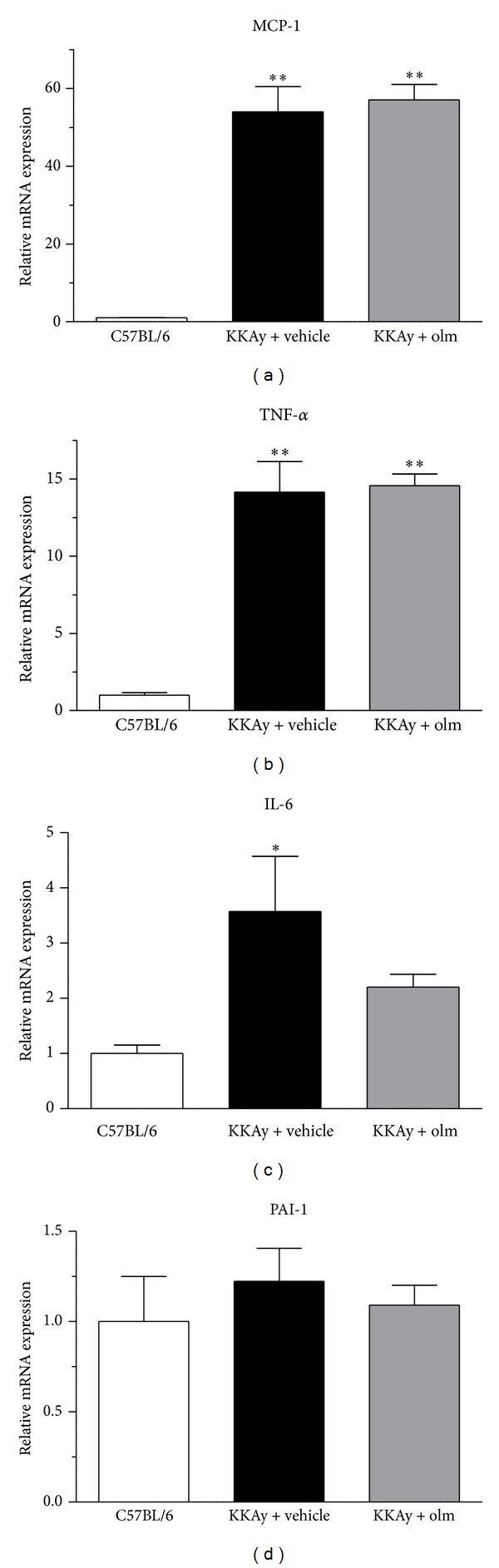
Effects of olmesartan (olm) on the adipose tissue mRNA expression of proinflammatory cytokines ((a) MCP-1; (b) TNF-*α*; (c) IL-6; and (d) PAI-1) in KKAy mice. The values are the mean ± SEM (*n* = 8). *P < 0.05, **P < 0.01 versus C57BL/6 (ANOVA). Olm indicates olmesartan.

Regarding the expression of tissue inflammatory cytokines (MCP-1, TNF-*α*, IL-6, and PAI-1) in adipose tissue (Figure 6), while the PAI-1 mRNA expression was not altered in the vehicle-treated KKAy mice (Figure 6(d)), the mRNA levels of MCP-1, TNF-*α*, and IL-6 were all significantly upregulated in the KKAy mice treated with vehicle compared with the control C57BL/6 mice (MCP-1 and TNF-*α*, *P* < 0.01; IL-6, *P* < 0.05 versus C57BL/6) (Figures 6(a), 6(b), and 6(c)). With respect to a possible effect of olmesartan on these inflammatory cytokine genes in the adipose tissue, the KKAy mice treated with olmesartan exhibited a blunted increase in adipose IL-6 mRNA expression (Figure 6(c)), in spite of the fact that there are no effects on the adipose MCP-1, TNF-*α*, and PAI-1 mRNA expression (Figures 6(a), 6(b), and 6(d)).

### 3.5. Effects of Olmesartan on Adipose Tissue Oxidative Stress

**Figure 7:**
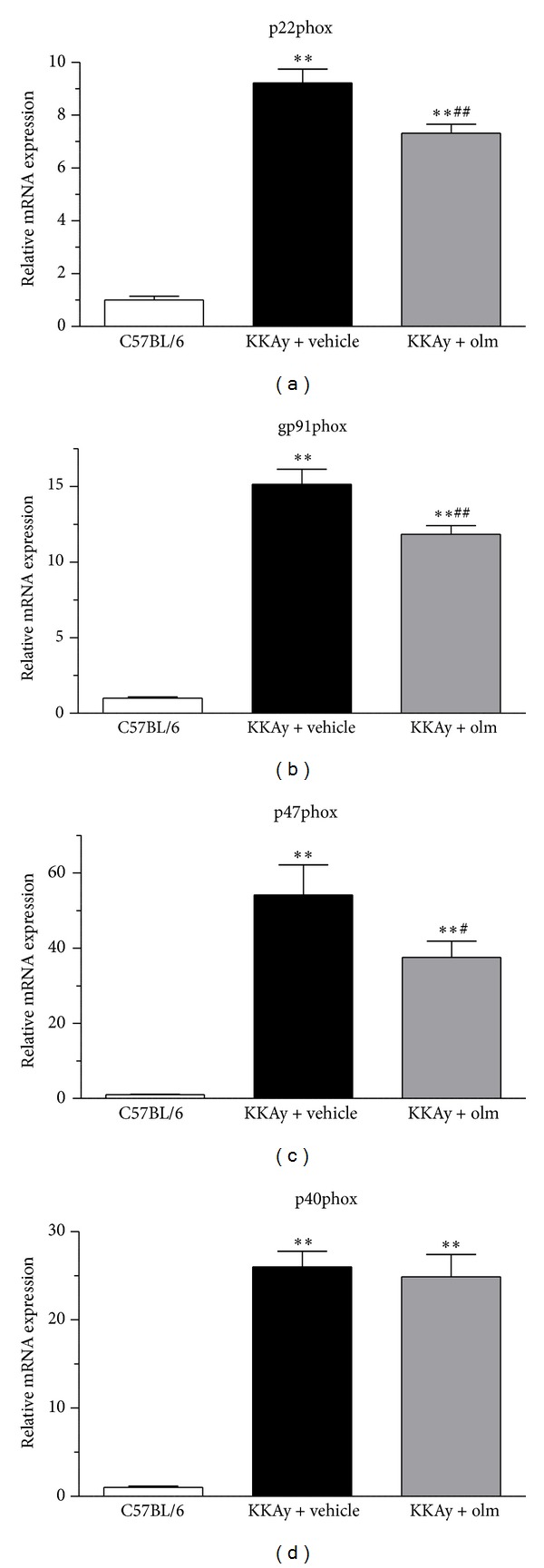
Effects of olmesartan (olm) on the adipose tissue mRNA expression of NADPH oxidase components ((a) p22phox; (b) gp91phox; (c) p47phox; and (d) p40phox) in KKAy mice. The values are the mean ± SEM (*n* = 8). **P < 0.01 versus C57BL/6; ^#^ *P* < 0.05, ^##^ *P* < 0.01 versus KKAy + vehicle (ANOVA). Olm indicates olmesartan.

We finally examined the possible effects of olmesartan on the expression of the NADPH oxidase components (p22phox, gp91phox, p47phox, and p40phox) in the epididymal adipose tissue of KKAy mice. As shown in Figure 7, although the vehicle-treated KKAy mice exhibited a significantly elevated expression of these NADPH oxidase component mRNA levels in the adipose tissue compared with the control C57BL/6 mice (p22phox, gp91phox, p47phox, and p40phox, *P* < 0.01 versus C57BL/6), treatment with olmesartan for 4 weeks significantly suppressed the enhanced adipose tissue expression of p22phox, gp91phox, and p47phox mRNA in KKAy mice without affecting adipose p40phox mRNA expression mice (p22phox and gp91phox, *P* < 0.01; p47phox, *P* < 0.05 versus vehicle) (Figures 7(a), 7(b), 7(c), and 7(d)).

## 4. Discussion

Increasing evidence has indicated that adipose tissue is profoundly involved in the physiological and pathophysiological regulation of circulatory and endocrine systems* in vivo* via modulatory effects on the local production of inflammatory cytokines, adipokines, and vasoactive factors. In addition, it has been demonstrated that the genes of RAS components such as angiotensinogen and AT1R were substantially expressed in adipose tissue [6]. The local RAS in adipose tissue is suggested to be critically involved in the modulation of the physiological function of adipocytes, and furthermore, the pathological activation of adipose tissue RAS reportedly plays a crucial role in the pathophysiology of metabolic disorders via the dysregulated production of oxidative stress, inflammatory cytokines, and adipokines in adipose tissue [7, 19]. Thus, it is certainly important to identify any beneficial effects of interventions on adipose tissue in order to develop a more efficient therapeutic strategy to treat metabolic disorders with obesity.

In the present study, 4-week olmesartan treatment significantly decreased blood pressure in KKAy mice, a human model of metabolic disorders, without any significant effects on dietary food intake or body weight gain. Thus, the hypotensive effect of olmesartan was exerted without any inhibitory effect on body weight gain in KKAy mice. However, from the point of view of possible pleiotropic effects of olmesartan on adipose tissue function, there are three interesting findings possibly related to adipose tissue in KKAy mice: (1) an inhibitory effect on adipocyte hypertrophy, (2) a suppressive effect on IL-6 gene expression, and (3) an ameliorating effect on oxidative stress.

Previous studies showed that the persistent low-grade activation of chronic inflammatory responses in adipose tissue plays an important role in the development of metabolic disorders with visceral obesity [20–26] and that chronic adipose tissue inflammation is provoked via the stimulated secretion of proinflammatory cytokines and factors derived from adipocytes [4, 27]. Although adiponectin is a well-established adipocyte-secreted endocrine factor involved in the pathophysiology of metabolic disorders and provides a functional link between adipose tissue and the immune system [5, 28], the circulating adiponectin level is reportedly decreased in metabolic disorders with visceral obesity [29]. In addition, PPAR*γ* is reported to improve adipocytokine dysregulation in adipose tissue, including adiponectin, in metabolic disorders [30]. In the present study, while the treatment of KKAy mice with olmesartan did not affect the adipose expression of adiponectin or PPAR*γ*, olmesartan inhibited the adipose tissue gene expression of IL-6, which is also one of the key players in the inflammatory process in adipose tissue in metabolic disorders [22, 31].

Accumulated adipose tissue-induced dysregulated production of adipocytokines, including proinflammatory cytokines such as IL-6, is reported to activate NADPH oxidase components [32–35]. Adipose NADPH oxidase-derived reactive oxygen species (ROS) function as important intracellular second messengers to activate many downstream signaling molecules that modulate endothelial function, pathological growth and migration of vascular cells, expression of pro-inflammatory mediators and modification of extracellular matrix [36–39]. All of these processes play important roles in the development of insulin resistance and cardiovascular disease in metabolic disorders with visceral obesity. In the present study, olmesartan exerted a suppressive effect on adipocyte hypertrophy concomitant with an inhibitory effect on the IL-6-oxidative stress axis without any body weight reducing effect in KKAy mice, a human model of metabolic disorders.

We previously identified ATRAP as a novel molecule interacting with AT1R and showed that ATRAP suppressed the Ang II-induced pathological responses of cardiovascular cells and tissues by promoting AT1R internalization [17, 18, 40–42]. Thus, a tissue-specific regulatory balancing of ATRAP and AT1R expression may be involved in the modulation of AT1R signaling that specifically occurs in each tissue [43–46]. We showed that the upregulation of the cardiac ATRAP/AT1R ratio is one of the therapeutic benefits of olmesartan in inhibiting cardiac hypertrophy in hypertensive rats [47]. In addition, prepubertal transient blockade of AT1R signaling by olmesartan exerted a long-term therapeutic effect on salt-induced hypertension and renal injury in Dahl Iwai salt-sensitive rats, partly through a sustained enhancement of renal ATRAP expression [48].

Furthermore, a recent study employing mice with the gene-targeted systemic deletion of ATRAP has shown that the development of systemic insulin resistance related to ATRAP deficiency is attributable to the exaggerated adipose tissue inflammation that occurs via the secretion of proinflammatory cytokines and factors derived from enlarged adipocytes, thereby suggesting ATRAP to be a novel molecular target in metabolic disorders in visceral obesity [19]. However, in the present study, the treatment with olmesartan exerted no evident influence on adipose tissue ATRAP gene expression in KKAy mice. Therefore, further studies are necessary to examine whether the adipose ATRAP is involved in the olmesartan-induced beneficial suppressive effect on the IL-6-oxidative stress axis in KKAy mice.

In the present study, the body weight did not differ significantly between the vehicle-treated and olmesartan-treated KKAy mice after 4 weeks. A previous study showed that olmesartan treatment at a dose of 10 mg/kg per day for 6 weeks exerted an inhibitory effect on body weight gain with a trend of reduction in adiposity without any evident change in food intake in obese Otsuka Long-Evans Tokushima Fatty rats [49]. In contrast, another study reported that treatment with regular chow containing 0.0015% olmesartan for 2 weeks resulted in a significant reduction in blood pressure level without any effect on food intake or body weight gain in KKAy mice [50]. These results suggest that the inhibitory effects of olmesartan on body weight and adipose tissue mass may depend on the diabetic animal model used and the condition of olmesartan treatment (dose and duration). Furthermore, as a limitation of the present study, although olmesartan significantly suppressed the enhanced adipose tissue mRNA expression of NADPH oxidase, this does not constitute direct evidence of an effect. Further studies on the protein expression of NADPH oxidase and adipose tissue production of oxidative stress are needed to obtain a definitive result.

## 5. Conclusions

In summary, the results of the present study in a mouse model of human metabolic disorders showed a therapeutic effect of olmesartan on adipose tissue in addition to its blood pressure lowering effect. The results suggest that the olmesartan-mediated inhibitory effect on adipocyte hypertrophy in KKAy mice is associated with a beneficial suppression of the IL-6-oxidative stress axis in adipose tissue. Further studies are needed to demonstrate this beneficial effect of olmesartan on adipose tissue oxidative stress and to clarify whether there is a functional role of adipose ATRAP in the pathophysiology of metabolic disorders.
